# Measuring situation awareness and team effectiveness in pediatric acute care by using the situation global assessment technique

**DOI:** 10.1007/s00431-019-03358-z

**Published:** 2019-03-21

**Authors:** Ester Coolen, Jos Draaisma, Jan Loeffen

**Affiliations:** 1 Department of Pediatrics (804), Radboud University Medical Centre Amalia Children’s Hospital, P.O. Box 9101, 6500 HB Nijmegen, The Netherlands; 2 Princess Maxima Centre for Pediatric Oncology, Utrecht, The Netherlands

**Keywords:** Situational awareness, Medical simulation, Teamwork, Crew resource management, Cardiopulmonary resuscitation, Pediatrics, Child

## Abstract

Situation awareness (SA) is an important human factor and necessary for effective teamwork and patient safety. Human patient simulation (HPS) with video feedback allows for a safe environment where health care professionals can develop both technical and teamwork skills. It is, however, very difficult to observe and measure SA directly. The Situation Global Assessment Technique (SAGAT) was developed by Endsley to measure SA during real-time simulation. Our objective was to measure SA among team members during simulation of acute pediatric care scenarios on the medical ward and its relationship with team effectiveness. Twenty-four pediatric teams, consisting of two nurses, one resident, and one consultant, participated in three acute care scenarios, using high-fidelity simulation. Individual SAGAT scores contained shared and complimentary knowledge questions on different levels of SA. Within each scenario, two “freezes” were incorporated to assess SA of each team members’ clinical assessment and decision-making. SA overlap within the team (team SA) was computed and compared to indicators of team effectiveness (time to goal achievement, consensus on primary problem, diagnosis, task prioritization, leadership, and teamwork satisfaction). In 13 scenarios (18%), the team failed to reach the primary goals within the prescribed time of 1200 s. There was no significant difference in failure of goal completion between the scripted scenarios; however, there was a significant difference between scenario 3 and the other scenarios in time to goal completion. In all three scenarios, SA overlap level 2 (consensus on primary problem during the first freeze and consensus on diagnosis during the second freeze) leads to significantly faster achievement of the predefined goals. There was a strong relationship between team SA on the primary problem and diagnosis and team SA on task prioritization. Consensus on leadership within the team was low. Teamwork satisfaction was more influenced by knowledge about the importance of the assigned task than outcome of the scenario.

*Conclusion*: The use of SAGAT enables us to measure SA of team members during real-time simulation of acute care scenarios. Although there is no direct connection between team SA and goal achievement, SAGAT provides insight in differences in SA among team members, and the process of shared mental model formation. By measuring SA, issues that may improve team effectiveness (prioritizing tasks, enhancing shared mental models, and providing leadership) can be trained and assessed during medical team simulation, enhancing teamwork in health care settings.

**Table:** 

**What is known?** • *Teamwork skills such as communication, leadership, and situational awareness have become increasingly recognized as essential for good performance in pediatric resuscitation. However, the assessment of pediatric team performance in these clinical situations has been traditionally difficult.* • *The Situation Awareness Global Assessment Technique (SAGAT) is a method of objectively and directly measuring SA during a team simulation using “freezes” at predetermined points in time with participants reporting on “what is going on” from their perspective on the situation.*	
**What is new?** • *We assessed SA, and its relationship with team effectiveness, in multidisciplinary pediatric teams performing simulated critical events in critically ill children on the medical ward using the SAGAT model, outside the emergency room setting.* • *In all three scenarios, consensus on the primary problem (shared mental model) leads to faster achievement of predefined goals. Consensus on leadership was overall low, without a significant impact on goal achievement.*

## Introduction

Life-threatening emergencies for children are rare and challenge pediatric care teams. Simulation team training programs provide a safe learning environment for pediatric teams dealing with these critically ill children [20, 31, 40]. Teamwork skills such as communication, leadership, and situational awareness have become increasingly recognized as essential components of crisis resource management (CRM) [19, 23, 27]. However, the assessment of pediatric team performance in clinical situations has been traditionally difficult.

There are currently few assessment tools that address the challenges in measuring team performance. Standard assessment tools in simulation are objective structured clinical examination (OSCE) checklists [34] and global rating scales of teamwork competencies (e.g., NOTSS, OCRMGRS) [1, 9, 25]. These approaches are questionable when it comes to assessing cognitive processes like clinical decision-making [37], especially when this is a team effort. Checklists put emphasis on results with minimal insight into the process. Therefore, this type of assessment may reward thoroughness rather than competence and may not allow for recognition of alternative approaches to the problem. Another part of the complexity of teamwork assessment lies in the difference between individual and team performance when it comes to human factor competencies like communication, leadership, decision-making, and situation awareness. Patient care in clinical settings is most often the result of a team effort as opposed to individual actions, e.g., technical skills, that can be assessed during skills stations. Studies conducted in both health and non-health care settings suggest that shared mental models (SMM) can help enhance team coordination and effectiveness [21, 28–30]. Furthermore, the degree to which team members share team mental models has been shown to correlate with team performance [24]. It follows then that health care professionals should not only be trained to recognize and adapt mental models, but also share and align them with other team members [13].

To gain insight into the cognitive processes that play a role in the selection of mental models and clinical decision-making by health care professionals during critical patient care, we need to introduce the concept of situational awareness (SA). This concept has been exported from military aviation to human factor engineering, and is defined by Endsley as “knowing what is going on around you” [11]. SA is subdivided into three levels of understanding; level 1 SA refers to perceptions of elements in the environment. This includes all information that appeals to the five senses: Examples in medical simulation are oxygen saturation, cardial pulse, blood pressure, and skin color. Level 2 SA involves the comprehension of level 1 factors. Team members build on data acquired during initial patient assessment. For example, a rapid heart rate and low blood pressure may indicate a circulation problem like hypovolemia. Level 3 SA is achieved when a team member makes projections based on understanding of level 1 and level 2 information and anticipates on events that may occur in the near future.

Endsley defines team SA as the degree to which each team member possesses the SA required for his or her responsibilities. Team members may have different subgoals in a given scenario but should work from the same shared mental model in any given situation. Overall team SA can be thought of as the degree to which every team member possesses the SA required for his or her responsibilities. This is independent of any overlap in SA requirements that may be present. If each of two team members needs to know a piece of information; it is not sufficient that one knows perfectly and the other does not [11]. It is not only the detailed situational information (data: level 1 SA) but also the way the pieces are put together (comprehension: level 2 SA) that direct decisions made within the team (projection: level 3 SA) [11]. So, it is important to realize that good SA can be viewed as a factor that will increase the probability of good performance but cannot guarantee it [11].

The Situation Awareness Global Assessment Technique (SAGAT) is a method of objectively and directly measuring SA designed by Endsley [12]. During a team simulation, the scenario is stopped at various predetermined points in time and participants complete written questions as to what is going on at that specific time. The scenario is then resumed and stopped again at predetermined points. Evidence supporting the SAGAT model has been established in environments including aviation, military, and transport [11]. SAGAT has also been validated in different medical domains especially in emergency, trauma, and obstetric teams [8, 16, 18, 33]. Hogan et al. compared individual SAGAT scores to level of training of the participant and their result showed statistically significant differences. These measurements provide evidence for construct validity of the SAGAT tool used in medical trauma scenarios designed in a similar fashion according to ATLS guidelines. The results of reliability analysis in the same study are also quite promising for SAGAT as an assessment tool. Analysis of the assessment technique including every “freeze” by every participant yielded a Cronbach’s alpha of 0.767. This value is over the threshold of 0.7, and acceptable for an assessment test [18].

However, the literature on the use of SAGAT for training and assessing team SA outside the emergency room is scarce. We therefore wished to expand and evaluate the use of the SAGAT assessment tool in medical team simulation to train and assess multidisciplinary pediatric teams performing simulated critical events in critically ill children.

## Methods

### Study design

Our study goals were to evaluate the use of the SAGAT assessment tool during multidisciplinary pediatric team training. In order to analyze team SA, we collected data on SA of individual participants during team simulation and compared overlap in SAGAT scores (consensus on the primary problem, diagnosis, task prioritization, and leadership) with time to goal achievement. Simulation team training is a part of professional training for all professionals in patient care at our hospital. For each scenario, there are clear designed goals, but also to each scenario content there are different sets of context information cards available and different complications can occur. In this way, we assure that participants will not know from the information given at the start of the scenario what will happen next. Also, participants are told not to disclose any information from the scenarios or debriefing to their colleagues to guarantee a safe and challenging learning experience. During the research period, we provided the same information to the participants. We changed the order of the scenarios for each training session and also changed context information irrelevant to the course of the scenario (e.g., name, age). Three pediatric critical care scenarios were developed and modified to add elements that would require complimentary knowledge and expertise by all team members. The setting in all three scenarios was a medium care pediatric ward, where the patient was already admitted and deteriorated shortly after the start of the scenario. During the three scenarios, participants were to recognize the primary problem of the patient according to the Airway, Breathing, Circulation, Disability, Exposure (ABCDE) approach and start the appropriate goal-directed treatment. For each scenario, a set of goals were designed according to the pediatric advanced life support (PALS) guidelines. The first scenario involved a 6-year-old child with an unwitnessed (neuro) trauma on the trampoline. Goals for this scenario were to recognize the signs and symptoms of increased intracranial pressure and need for decompression neurosurgery. The second scenario involved a 3-year-old girl presenting with signs of dehydration due to a rotavirus gastroenteritis, deteriorating after rehydration due to ischemic bowel obstruction (volvulus). Goals were recognition of the signs of small-bowel obstruction (bilious vomiting) and ischemia (pain and elevated lactate) and start fluid resuscitation by administering two boluses of crystalloids. The third scenario involved a 4-year-old boy recovering after abdominal surgery showing signs of an anaphylactic reaction to a blood transfusion. Goals were to identify hypotension and rash, stop all ongoing infusions, and administer epinephrine intramuscularly. The scenarios were programmed into a human patient simulator (HPS (Pediasim®)). The scenarios were run in a simulation unit consistent with a normal pediatric medium care wardroom. It was equipped with materials, monitors, and instruments that will normally be present in a medium care patient room. All three scenarios were designed to challenge SA and promote the need for team awareness to make adequate decisions. Parts of essential information was given to different participants, and there were alterations in workload and distracting elements (telephone, non-essential info, etc.). The two pediatric nurses who started the scenario together were able to call the pediatric resident at any time they saw fit, but no later than 5 min after the start of the scenario. The first freeze appeared after 10 min (before arrival of pediatric consultant). The second freeze was at the end of the scenario after 20 min. The maximum duration of a freeze was 3 min. SA queries were developed using goal-directed task analysis as described by Endsley [12]. During the first freeze, all participants were asked to answer a total of eight MCQ (multiple choice) questions on level 1 (e.g., oxygen saturation), level 2 (e.g., primary problem), and level 3 (e.g., first task to be executed) of SA on a hand-held device (tablet). During the second freeze, at the end of the scenario, participants were asked again to answer 8 MCQ questions on levels 1 to 3 of SA. Moreover, they had to indicate the suspected diagnosis (level 2 SA), who they perceived to be the team leader at the end of the scenario, and were asked to rate their satisfaction with teamwork on a Likert scale from 1 to 10 (1: very poor teamwork, 10: excellent teamwork). Examples of MCQ questions at different levels of SA are given in Table 1. An overview of SAGAT queries for all three scenarios can be found in the Appendix. Using retrospective video review, team effectiveness was measured by scoring the time when the pre-designed goals of the scenario were completed by the team. When the team did not accomplish these pre-designed goals within 20 min (1200 s), this was viewed as a failure of goal completion. After each simulated scenario session, a video-assisted debriefing took place, led by an experienced facilitator focusing on teamwork skills.

**Table 1:** Example SAGAT participant query format

SAGAT participant query format	
Level 1 *Data*	
What is the patient’s oxygen saturation?	
a. > 95%	
b. 90–95%	
c. < 90%	
Level 2 *Comprehension*	
What is the primary problem of the patient?	
a. Airway	
b. Breathing	
c. Circulation	
d. Disability	
Level 3 *Projection*	
Which treatment needs to be prioritized at this point?	
a. Mask/bag ventilation	
b. Fluid challenge	
c. Epinephrine i.m.	
d. Surgery	
What is the suspected diagnosis of the patient’s condition?	

### Participants

During the period of 1 year (November 2015 until November 2016), 24 pediatric professional teams of the Radboudumc Amalia Children’s hospital participated in multidisciplinary simulation training sessions each completing 3 scenarios in a random order. A pediatric team was composed of two pediatric nurses, 1 pediatric resident, and 1 pediatric consultant.

The simulated scenarios were designed and teams were composed in order to match the actual clinical situation at the medium ward in our hospital. When a monitored patient admitted to the medium care ward would deteriorate, the responsible nurse would be the first person to detect a change in vitals and could call for help from a second nurse in order to assess the patient. When a change in the patient condition is assessed by the nurses, the attending resident is contacted and joins the team to evaluate and stabilize the patient. At any given time, as was the case in the scenarios, the resident can consult with the supervising pediatrician for support; however, the consultant would not arrive at the scene until 10 min into the scenario as would be realistic in the clinical context. In this way, we realistically simulated the clinical situation in order to assess the building of a shared mental model and level of situational awareness at different points during the scenario. Before the start of the first scenario, all team members were given a description of the study and a short orientation to the HPS and the simulation room. They were instructed about the random freezes and to answer the SAGAT questions individually in a room next to the simulation unit. Informed written consent and permission to record and store video data of the scenarios for the purpose of research was obtained from all participants. At the onset of the scenario, one of the nurses and resident were separately given a structured patient handover by the facilitator simulating an attending colleague at the end of his shift that introduced them to the simulated patient’s relevant history and status. All study participants completed the three scenarios and filled out the individual SAGAT scores during the scenarios. Twenty-four residents, 24 consultants, and 48 nurses completed 72 scenarios. During each scenario, 3 SAGAT scores (2 nurses and 1 resident) were collected and compared during the first freeze and 4 SAGAT scores were collected and compared during the second freeze when the consultant was also part of the pediatric team.

### Statistical analysis

Data are presented as mean (SD) and/or median as appropriate. We used the Fisher’s exact test to compare proportions of a categorical outcome according to different independent groups and the Kruskal–Wallis one-way analysis of variance for non-parametric distributed data. Statistical analyses were performed using SPSS version 22.0. A *p* value < 0.05 was considered statistically significant.

## Results

### Situational awareness and goal achievement

The mean time to goal completion was 965 s, with a standard deviation of 162 s (Table 2). In 13 scenarios (18%), the team failed to reach the predefined goals within the prescribed time of 1200 s. There was no significant difference in failure of goal completion between the scripted scenarios; however, there was a significant difference between scenario 3 and the other scenarios in time to goal completion (Table 2). Fisher’s exact test showed no statistical difference between teams with or without consensus on the primary problem (ABCD) and goal achievement (*p* = 0.359). Consensus on diagnosis at the end of the scenario and goal achievement within the prescribed time shows a trend toward a positive association, although it did not reach a statistical difference (*p* = 0.059). Furthermore, we found a strong association between consensus on the primary problem (ABCDE) within the team during the first freeze and consensus on diagnosis at the end of the scenario during the second freeze (*p* = 0.000). The same strong relationship was found for consensus on the primary problem and consensus on task prioritization within the team (*p* = 0.001).

**Table 2:** Mean time to goal achievement in seconds and number of goal achievements and failure to completion per scenario, with significant faster goals achievement in scenario 1 and 2 in comparison with scenario 3 (*p = 0.02)

Scenario	Mean (sec)	Goal achievement (*N*)	Std. deviation (sec)	Failure to complete goal (*N*)
1.Neurotrauma	938.26	19	165.34	5
2. Volvulus	894.86	22	150.00	2
3. Anaphylaxis	1079.11*	18	110.49	6
Total	965.05	59	162.32	13

**Fig. 1:**
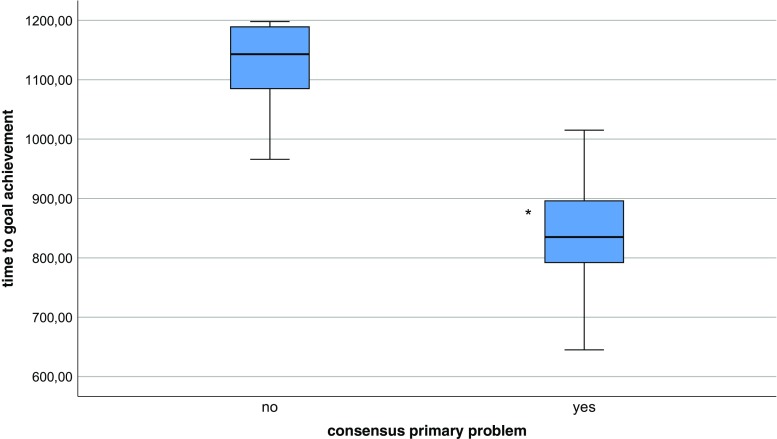
Q-Q plot. Association between team consensus on primary problem (ABCDE) and time to goal achievement. * Significant difference among groups (*p* < 0.05)

**Fig. 2:**
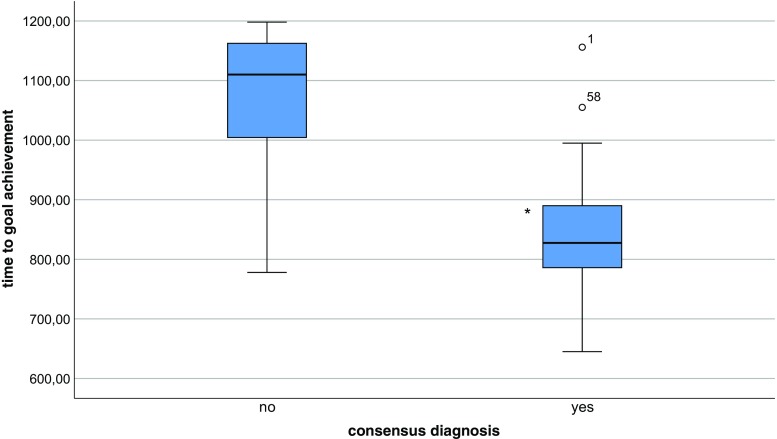
Q-Q plot. Association between team consensus on diagnosis (patient condition) to goal achievement. * Significant difference among groups (*p* < 0.05)

In all three scenarios, SA overlap level 2 (consensus on the primary problem during the first freeze and consensus on diagnosis during the second freeze) faster achievement of the predefined goal was accomplished (Figs. 1 and 2).

### Situational awareness and task prioritization

**Fig. 3:**
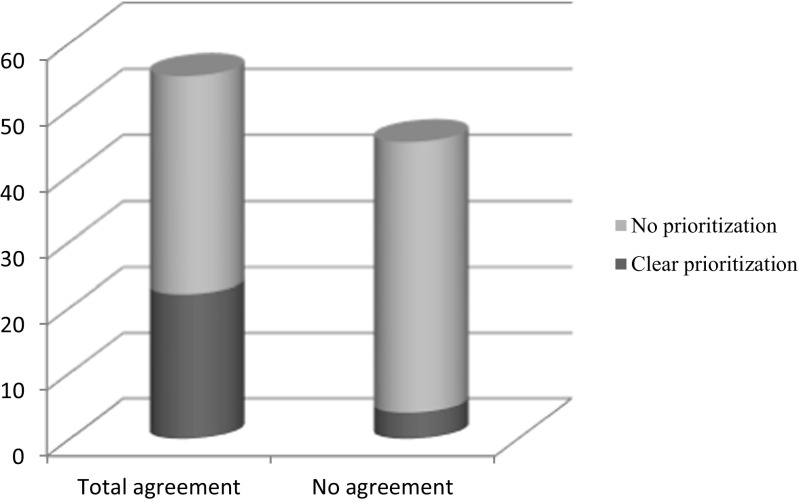
Relationship between team agreement on primary problem and consensus on task prioritization

In 40 scenarios (56%), there was a 100% agreement on the primary problem of the patient (ABCD). In 20 scenarios (28%), there was a 100% agreement on primary task to be executed: There is a strong relationship between team SA (100% agreement within the team) on the primary problem and team SA on task prioritization: 80% of team that had a 100% agreement on task prioritization (level 3 SA) also had 100% agreement on the first task to be executed (level 2 SA) at the time of the first freeze (Fig. 3).

### Situational awareness and leadership

**Fig. 4:**
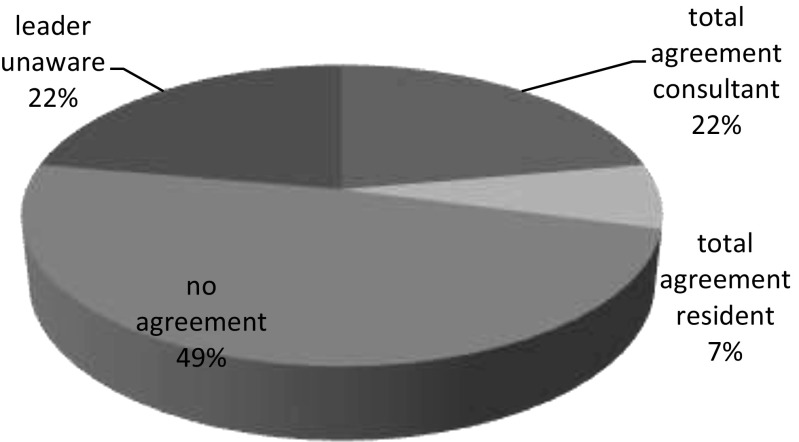
Leadership awareness of team members at the scenario (second freeze)

In 21 scenarios (29%), there was 100% agreement on leadership. When there was 100% agreement among team members, this was more often the case when the consultant was perceived to be the leader (76% consultant versus 24% resident). In 16 scenarios (22%), the leader was not aware of his own leadership at the time (Fig. 4).

### Teamwork satisfaction and goal achievement

Participants rated their satisfaction with teamwork, during the second freeze at the end of the scenario, but before debriefing the scenario. The mean overall score on teamwork satisfaction was 7.27 (median 7.25). There was no significant difference between participant satisfaction with teamwork and achieving or failing the predefined goals (7.15 versus 7.30). A total of 13 participants rated teamwork a 6 or below (18% of participants; 9 times nurse 2 in the scenario, 4 times the resident). Deviant scores were discussed during video-debriefing with the nurses indicating that low scores were given when they had to execute tasks without feedback from the team leader, and the residents explained that they were more negative about teamwork when there was no consensus on team leadership when the consultant entered the room.

## Discussion

A number of studies have demonstrated an improvement in outcomes following simulated team training [7, 10, 26]. However, assessment of team performance is challenging and requires consideration of multiple components of teamwork. Several studies on medical team efficiency have suggested deficiencies in team SA [6, 32, 35] and support our development of a SAGAT educational tool for pediatric team training for decision-making in critical clinical situations. In pediatric simulation, research is done on assessment of communication and leadership skills [3, 15, 36], but none has primary focused on team SA. We have developed scenario training using the SAGAT educational tool to assess team performance during critical clinical situations, incorporating the vital construct of SA. Individual and team SA is crucial to proper team function in dynamic environments where errors in perception, comprehension, and projection can lead to negative outcomes in patient care [11, 41]. Measurement of team SA by monitoring overlap in individual SA using the SAGAT tool during a HPS scenario provides insight into the decision-making process of pediatric teams, which could influence models for health care setting team training and curriculum development and actual clinical performance [35].

### Situation awareness: goal achievement and task prioritization

Higher rate of SA overlap (overall) does not automatically lead to faster achievement of the predefined goals. Physiological cues and perception of monitor signs (level 1 SA) were not associated with faster goal achievement in any scenario and often there was no consensus on these cues within the team (data not shown). For example, there was only 45% team agreement on level I SA (e.g., respiratory rate, oxygen saturation, color of gastric fluid). Although these misconceptions do not lead to a measurable difference in goal achievement, these findings are of concern, because monitoring and repeated assessment of vital signs are important and extensively trained skills to both nurses and physicians, whereas they can predict possible deterioration of the patient in the near future. Despite educational effort to train nurses and physicians in continuous vital sign surveillance to improve patient safety, this information often is not shared within the team, possibly leading to suboptimal teamwork and patient care. On the other hand, we were able to detect a strong relationship between team consensus on the primary problem in the first part and diagnosis in the second part of the scenario (level 2 SA) and also with team consensus on task prioritization (level 3 SA). This is in line with a study measuring clinical performance in nursing students. Higher SA scores on level II and III correlated with higher rated performance in a OSCE Shock scenario when teams were assessed using a standard score sheet [2]. A better understanding of the patient’s problem by all team members seems to enhance effectiveness of teamwork in division and prioritizing tasks at hand. Teams with 100% SA overlap level 2 (diagnosis), meaning all team members shared the same mental model at the time of the first freeze, were significantly faster in achieving the predefined goals of the scenario. This supports our hypothesis that a shared mental model directs decision-making and leads to better information integration and prediction of future events [11], thereby enhancing effective teamwork and goal achievement. Team consensus on the primary problem was a very good predictor of team consensus on diagnosis in the second part of the scenario. This emphasizes the importance of a (ABCDE) structured approach of the deteriorating child, with clear communication within the team about the problem at hand and the sequence of actions to be performed. However, the impact of consensus on the primary problem was not the same for all three scenarios. In our ischemic bowel scenario, nearly all teams (22 out of 24 teams) achieved the scenario-specific goals within the prescribed time of 1200 s, regardless of the level of consensus on the patient condition within the team (data not shown). This could be explained by the fact that it was difficult for the pediatric teams to be certain of the diagnosis ischemic bowel (volvulus), whereas dehydration and sepsis fit the same pattern of symptoms and action to treat the patient (start fluid resuscitation by administering two fluid boluses of crystalloids). When the diagnosis is not shared within the team, members could have a different mental model in mind and still start the right action to reach the appropriate goal, as could also be the case in clinical practice. Therefore, in order to design a simulation scenario, underlining the importance of building a shared mental model within the team, one should bear in mind that different mental models should lead to different actions in order to have an effective training experience. We found a significant difference in mean time to goal achievement between the anaphylaxis scenario, compared to the other two scenarios, with the same distribution of team consensus. Teams took longer to complete the third scenario regardless of their level of shared situational awareness. Our hypothesis is that for this specific scenario, we designed a complex patient after abdominal surgery. Therefore, it was difficult for the team to decide whether this was a typical case of anaphylaxis. Vital parameters could also fit the pattern of peritonitis with sepsis and/or perforation. The primary goals were to stop transfusion and give adrenaline i.m. However, both nurses and physicians hesitated to give adrenaline before fluids and antibiotics were given to the patient. It could be argued that, because giving adrenaline to a patient is not part of daily care at the medium care, there is a higher threshold to administer this to a patient and participants lean toward prioritizing actions they are more familiar with on a daily basis like administering fluids or antibiotics. This shows that, despite the great effort we made, it is very difficult to design equally challenging team scenarios to assess team SA.

In 13 scenarios (18%), the team failed to reach the predefined goals within 20 min. Outside of this particular training situation, we would have opted to provide the team with more clues in order to have a successful experience. In the simulation education community, some controversy exists with aspect to the emotional and educational consequences of mannequin deterioration or death due to trainees’ actions or inaction. Death of a simulated patient decreases self-efficacy in resuscitation skills of participants [39]. However, debriefing was found to significantly reduce negative emotions and deepen the learning experience [4, 38].

### Situational awareness and leadership

Leadership has been identified as a key variable for the functioning of teams and as one of the main reasons for success or failure of team-based work systems. Teams need to be organized in such a way that the individual skills of the team members can be used efficiently and effectively. It has been shown that failure to establish leadership for critical care teams can cause suboptimal teamwork and therefore increased risk to patients [5, 17]. We were surprised to find that in almost one half of the team scenarios (49%), there was no leadership agreement within the team. This could seriously impair team function and SA. In 22% of scenarios, the appointed team leader was not aware of his function. This was mostly the case when the consultant entered the room and joined the team without discussing leadership. During video observation of the scenarios, we were able to observe that sometimes the team automatically decided, without debate, that the consultant would become the leader because of seniority; other times, the resident continued to be the unspoken leader, because of actions already being put in motion. In scenarios with unanimity of leadership within the team, the consultant entered the room, asking immediate attention from the team and explicitly deciding on leadership. For the resident, it seemed to be more difficult to be the team leader to all team members, probably because of the implicit assumption of hierarchy being more important to nurses than the person with best situation overview when it comes to leadership. Nurses indicated during debriefing that they were often confused when leadership roles were not explicitly divided in control and (re) assessment function (hands-on leadership) and overview function (hands-off leadership).

### Teamwork satisfaction and goal achievement

We were surprised to find that there is no significant difference between participant satisfaction with teamwork and achieving or failing the predefined goals of the scenario, as we would have expected that a successful patient outcome would have a positive effect on team experience. We focused on participant rating teamwork lower than other team members to find out more their rationale. Low scores were almost exclusively given by the second nurse in the room (with the least patient information). Discussing team scores after video-debriefing taught us that executing tasks without sharing the mental model or feedback on the importance or performance of the tasks has a negative impact on motivation and team experience. In some cases, it was the resident feeling insecure about their performance when the consultant took over leadership position without deliberation. This underlines the theory that an effective leader should not only divide tasks, but also evaluate and adjust task execution within the team [22]. Our observations and teamwork satisfaction scores indicate that individual team satisfaction does not merely depend on patient outcome. Every team member needs to be informed about the mental model of the patient situation and actions to be taken. Every team member needs to receive feedback on the tasks they are executing on how this fits in the bigger picture of the situation at hand. Team members need to share information and alternatives to create a shared mental model and a goal-directed plan of action.

#### Strengths

Our study provides insight in real-time differences between individual SA versus team SA and the construction of shared mental models within a pediatric care team. Our main strength was the use of standardized simulation scenarios and the validated SAGAT tool to measure team SA and its relation to goal achievement in pediatric resuscitation. Whereas the pediatric resuscitation is most often the result of a team effort, we made an important effort to gain more insight into not only the cognitive processes that play a role in the selection of mental models and clinical decision-making by measuring outcome-related results (e.g., goal achievement, survival, team satisfaction), but also the formation of a shared mental model by measuring real-time SA during simulated pediatric resuscitation and its effect on team effectively. These results help us to better understand and improve teamwork during acute pediatric care.

#### Limitations

We consolidated the SAGAT scores into a team SA overlap score to represent “team” SA. This is just one approach and other approaches may be entertained. Other studies have measured individual SAGAT scores during a team effort and calculate TSAGAT as the sum of each team member’s individual score (both shared and complimentary knowledge) [8, 14]. In this way, you can measure the amount of information that is present within the team and correlate this with goal achievement. In our study, we wanted to gain insight in the information sharing process and development of shared mental models. That is why we decided to focus on shared SA within the team and its effect on team effectiveness. In addition, the process of defining SAGAT questions is context and discipline specific and cannot be directly translated to other fields of clinical practice. Another limitation of this study is the fact that we did not evaluate team performance during real-life resuscitation to determine whether the learning that occurred translated into better team performance during clinical practice. However, we invested in the design of very realistic scenarios, representative of the pediatric medium care ward, using a real-time high-fidelity simulation manikin. The interior of the room, available equipment, and team composition and task description were similar to the actual clinical setting. We are confident that these highly realistic conditions ensure that our findings on teamwork and goal achievement can be translated to the daily clinical practice. Furthermore, because of the standardized study setting, we paused the scenario after 10 min for the first freeze, restarting the scenario in the presence of the consultant. After a total of 20 min, the scenario stopped, defining goal achievement within this time frame as a success and non-achieving the primary scenario goals as failure, regardless of the condition of our simulated patient. We choose these time frames, because they match a realistic time course in clinical practice with the primary survey of the patient by nurses and resident and start of actions in the first 10 min and the consultant arriving thereafter, re-evaluating the patient and establishing a shared mental model, diagnoses, and action plan within the team.

## Conclusions

The use of SAGAT is feasible in medical team simulation and enables us to measure SA of team members during real-time simulation of acute care scenarios. Although we could not find a direct relationship between overall team SA and goal achievement, SAGAT provides insight in real-time differences between individual SA versus team SA and the construction of a shared mental models within the team. By measuring real-time team SA, issues that may improve team effectiveness (prioritizing tasks, enhancing shared mental models, and providing leadership) can be trained and assessed during medical simulation. If teams are trained and assessed together, and discuss differences in awareness of the situation, they will likely perform better in real clinical practice. Future studies should focus on the transfer of these human factor competencies from simulation to clinical practice.

## Appendix

**Table:**

SAGAT participant query format scenario 1A (first freeze)	
Level 1 *Data*	
1. Please state your assessment of the patient’s airway patency:	
a. Open	
b. Threatened	
c. Obstructed	
d. I do not know	
2. Please state your assessment of the patient’s work of breathing:	
a. Normal work of breathing	
b. Increased work of breathing	
c. Near respiratory failure	
d. I do not know	
3. Please state your assessment of the patient’s oxygen saturation:	
a. > 95%	
b. 90–95%	
c. < 90%	
d. I do not know	
4. Please state your assessment of the patient’s circulation:	
a. Normal circulation	
b. Decreased circulation	
c. Near circulatory failure	
d. I do not know	
5. Please indicate the systolic blood pressure of the patient:	
a. < 95 mmHg	
b. 95–120 mmHg	
c. > 120 mmHg	
d. I do not know	
6. Please state your assessment of this patient’s consciousness:	
a. Alert	
b. Responsive to verbal stimuli	
c. Responsive to pain	
d. Unresponsive	
e. I do not know	
7. Please state your assessment of the patient’s pupils:	
a. Pupils constricted	
b. Pupils normal, responsive to light	
c. Unilaterally dilated	
d. I do not know	
8. What is the patient’s HB count?	
a. < 5 mmol/L	
b. 5–7 mmol/L	
c. > 7 mmol/L	
d. I do not know	
Level 2 *Comprehension*	
1. What is the primary problem of the patient?	
a. Airway	
b. Breathing	
c. Circulation	
d. Disability	
e. I do not know	
Level 3 *Projection*	
1. Which action needs to be prioritized at this point?	
a. Mask/bag ventilation	
b. Fluid challenge	
c. CT-scan or MRI	
d. Surgery	
2. Is the team complete at this point or do other professionals need to be notified? If so which professional needs to be included in the team?

**Table:**

SAGAT participant query format scenario 1B (second freeze)	
Level 1 *Data*	
1. Please state your assessment of the patient’s airway patency:	
a. Open	
b. Threatened	
c. Obstructed	
d. I do not know	
2. Please state your assessment of the patient’s work of breathing:	
a. Normal work of breathing	
b. Increased work of breathing	
c. (Near)respiratory failure	
d. I do not know	
3. Please state your assessment of the patient’s oxygen saturation:	
a. > 95%	
b. 90–95%	
c. < 90%	
d. I do not know	
4. Please state your assessment of the patient’s circulation:	
a. Normal circulation	
b. Decreased circulation	
c. (Near) circulatory failure	
d. I do not know	
5. Please indicate the systolic blood pressure of the patient:	
a. < 95 mmHg	
b. 95–120 mmHg	
c. > 120 mmHg	
d. I do not know	
6. Please state your assessment of this patient’s consciousness:	
a. Alert	
b. Responsive to verbal stimuli	
c. Responsive to pain	
d. Unresponsive	
e. I do not know	
Level 2 *Comprehension*	
1. Please state your diagnosis (most important problem to treat for this patient):	
Level 3 *Projection*	
1. Who is the team leader at this point?	
2. Please state your assessment of overall team function (0–10)

**Table:**

SAGAT participant query format scenario 2A (first freeze)	
Level 1 *Data*	
1. Please state your assessment of the patient’s airway patency:	
a. Open	
b. Threatened	
c. Obstructed	
d. I do not know	
2. Please state your assessment of the patient’s work of breathing:	
a. Normal work of breathing	
b. Increased work of breathing	
c. (Near) respiratory failure	
d. I do not know	
3. Please state your assessment of the patient’s oxygen saturation:	
a. > 95%	
b. 90–95%	
c. < 90%	
d. I do not know	
4. Please state your assessment of the patient’s circulation:	
a. Normal circulation	
b. Decreased circulation	
c. (Near) circulatory failure	
d. I do not know	
5. Please indicate the systolic blood pressure of the patient:	
a. < 65 mmHg	
b. 65–95 mmHg	
c. > 95 mmHg	
d. I do not know	
6. Please state your assessment of this patient’s consciousness:	
a. Alert	
b. Responsive to verbal stimuli	
c. Responsive to pain	
d. Unresponsive	
e. I do not know	
7. What is the result of the venous lactate measurement in this patient?	
a. < 2 mmol/L	
b. 2–5 mmol/L	
c. > 5 mmol/L	
d. I do not know	
8. Please state your assessment of the patient’s gastric fluid retention?	
a. Clear aspect	
b. Green, bilious aspect	
c. Red, bloody aspect	
d. I do not know	
Level 2 *Comprehension*	
1. What is the primary problem of the patient?	
a. Airway	
b. Breathing	
c. Circulation	
d. Disability	
e. I do not know	
Level 3 *Projection*	
1. Which action needs to be prioritized at this point?	
a. Mask/bag ventilation	
b. Fluid challenge	
c. CT-scan or MRI	
d. Surgery	
e. I do not know	
2. Is the team complete at this point or do other professionals need to be notified? If so which professional needs to be included in the team?

**Table:**

SAGAT participant query format scenario 2B (second freeze)	
Level 1 *Data*	
1. Please state your assessment of the patient’s airway patency:	
a. Open	
b. Threatened	
c. Obstructed	
d. I do not know	
2. Please state your assessment of the patient’s work of breathing:	
a. Normal work of breathing	
b. Increased work of breathing	
c. (Near) respiratory failure	
d. I do not know	
3. Please state your assessment of the patient’s oxygen saturation:	
a. > 95%	
b. 90–95%	
c. < 90%	
d. I do not know	
4. Please state your assessment of the patient’s circulation:	
a. Normal circulation	
b. Decreased circulation	
c. (Near) circulatory failure	
d. I do not know	
5. Please indicate the systolic blood pressure of the patient:	
a. < 65 mmHg	
b. 65–95 mmHg	
c. > 95 mmHg	
d. I do not know	
6. Please state your assessment of this patient’s consciousness:	
a. Alert	
b. Responsive to verbal stimuli	
c. Responsive to pain	
d. Unresponsive	
e. I do not know	
Level 2 *Comprehension*	
1. Please state your diagnosis (most important problem to treat for this patient):	
Level 3 *Projection*	
1. Who is the team leader at this point?	
2. Please state your assessment of overall team function (0–10)

**Table:**

SAGAT participant query format scenario 3A (first freeze)	
Level 1 *Data*	
1. Please state your assessment of the patient’s airway patency:	
a. Open	
b. Threatened	
c. Obstructed	
d. do not know	
2. Please state your assessment of the patient’s work of breathing:	
a. Normal work of breathing	
b. Increased work of breathing	
c. (Near) respiratory failure	
d. I do not know	
3. Please state your assessment of the patient’s oxygen saturation:	
a. > 95%	
b. 90–95%	
c. < 90%	
d. I do not know	
4. Please state your assessment of the patient’s circulation:	
a. Normal circulation	
b. Decreased circulation	
c. (Near) circulatory failure	
d. I do not know	
5. Please indicate the systolic blood pressure of the patient	
a. < 65 mmHg	
b. 65–100 mmHg	
c. > 100 mmHg	
d. I do not know	
6. Please state your assessment of this patient’s consciousness:	
a. Alert	
b. Responsive to verbal stimuli	
c. Responsive to pain	
d. Unresponsive	
e. I do not know	
7. What infusions are actively administered to this patient at this point?	
a. Packed cells	
b. Morphine	
c. Potassium chloride	
d. None active infusions	
e. I do not know	
8. Please state your assessment of the patient’s abdominal wound?	
a. Dry aspect, no sign of infection	
b. Pus discharge from wound	
c. Bloody discharge from wound	
d. Redness, no discharge	
e. I do not know	
Level 2 *Comprehension*	
1. What is the primary problem of the patient?	
a. Airway	
b. Breathing	
c. Circulation	
d. Disability	
e. I do not know	
Level 3 *Projection*	
1. Which action needs to be prioritized at this point?	
a. Mask/bag ventilation	
b. Fluid challenge	
c. Stop all infusions	
d. Adrenaline i.m.	
e. I do not know	
2. Is the team complete at this point or do other professionals need to be notified? If so which professional needs to be included in the team?

**Table:**

SAGAT participant query format scenario 3B (second freeze)	
Level 1 *Data*	
1. Please state your assessment of the patient’s airway patency:	
a. Open	
b. Threatened	
c. Obstructed	
d. I do not know	
2. Please state your assessment of the patient’s work of breathing:	
a. Normal work of breathing	
b. Increased work of breathing	
c. (Near) respiratory failure	
d. I do not know	
3. Please state your assessment of the patient’s oxygen saturation:	
a. > 95%	
b. 90–95%	
c. < 90%	
d. I do not know	
4. Please state your assessment of the patient’s circulation:	
a. Normal circulation	
b. Decreased circulation	
c. (Near) circulatory failure	
d. I do not know	
5. Please indicate the systolic blood pressure of the patient:	
a. < 75 mmHg	
b. 75–100 mmHg	
c. > 100 mmHg	
d. I do not know	
6. Please state your assessment of this patient’s consciousness:	
a. Alert	
b. Responsive to verbal stimuli	
c. Responsive to pain	
d. Unresponsive	
e. I do not know	
Level 2 *Comprehension*	
1. Please state your diagnosis (most important problem to treat for this patient):	
Level 3 *Projection*	
1. Who is the team leader at this point?	
2. Please state your assessment of overall team function (0–10)	

## Abbreviations

SA: Situational awareness
HPS: Human patient simulator
SAGAT: Situation Global Assessment Technique
CRM: Crew resource management
OSCE: Objective structured clinical examination
NOTSS: Non-technical skills for surgeons
OCRMGRS: Ottawa Crisis Resource Management Global Rating Scale
SMM: Shared mental model
ABCDE: Airway, Breathing, Circulation, Disability, Exposure
PALS: Pediatric advanced life support
MCQ: Multiple-choice test
